# Transcriptomic Profiles of Splenic CD19^+^ B Cells in Mice Chronically Infected With the Larval *Echinococcus granulosus*

**DOI:** 10.3389/fvets.2022.848458

**Published:** 2022-04-25

**Authors:** Shiping Xu, Yuxin Guo, Tiancheng Luo, Pengfei Jiang, Ziyi Yan, Yan He, Linlin Fu, Hua Liu, Zixuan Gao, Dingmin Wang, Zhengxiu Sun, Xiaoying Yang, Wei Pan, Fenfen Sun

**Affiliations:** ^1^Jiangsu Key Laboratory of Immunity and Metabolism, Department of Pathogen Biology and Immunology, Xuzhou Medical University, Xuzhou, China; ^2^The First Clinical Medical College, Xuzhou Medical University, Xuzhou, China; ^3^National Experimental Teaching Demonstration Center of Basic Medicine (Xuzhou Medical University), Xuzhou, China; ^4^National Institute of Parasitic Diseases, Chinese Center for Disease Control and Prevention (Chinese Center for Tropical Diseases Research), Shanghai, China; ^5^National Health Commission Key Laboratory of Parasite and Vector Biology, Shanghai, China; ^6^World Health Organization Collaborating Centre for Tropical Diseases, Shanghai, China; ^7^National Center for International Research on Tropical Diseases, Shanghai, China; ^8^Department of Physiology, Xuzhou Medical University, Xuzhou, China

**Keywords:** *Echinococcus granulosus*, protoscoleces, B cells, immune regulation, metabolic reprogramming, lipid metabolism

## Abstract

**Background:**

We previously reported that the larval *Echinococcus granulosus* (*E. granulosus*) infection can expand the population of regulatory B cells in mice, thereby inhibiting the anti-infective immunity. However, the underlying mechanism is still largely unknown. This study further investigated the holistic transcriptomic profiles of total splenic B cells following the chronic infection of the parasite.

**Methods:**

The infection model of larval *E. granulosus* was established by intraperitoneal inoculation with 2000 protoscolexes. Magnetic-Activated Cell Separation (MACS) was used to isolate the total splenic B cells. RNA sequencing was performed to screen the differentially expressed genes (DEGs) after infection. The expression of selected DEGs was verified using qRT-PCR. Gene Ontology (GO) analysis, Kyoto Encyclopedia of Genes and Genomes (KEGG) pathway analysis, and Co-expression network analysis were applied to predict these DEGs' underlying biological processes, pathways, and interactions respectively.

**Results:**

A total of 413 DEGs were identified in larval *E. granulosus* infected B cells, including 303 up- and 110 down-regulated genes. Notably, most DEGs related to inflammation and chemotaxis were significantly upregulated after infection. In line with these changes, significant expression upregulation of DEGs associated with fatty acid oxidation, lipid synthesis, lipolysis, lipid transport, and cholesterol biosynthesis, were observed in infected B cells. Co-expression network analysis showed an intimate interaction between these DEGs associated with immune and metabolism.

**Conclusions:**

The present study revealed that the larval *E. granulosus* infection induces metabolic reprogramming of B cells, which provides a novel clue to clarify the immunoregulatory mechanism of B cells in parasitic infection.

## Introduction

*Echinococcus granulosus* (*E. granulosus*) is one of the cestodes that cause cystic hydatid disease, which poses a serious risk for public health and economic development (1). The distribution of the parasite is endemic and it is frequently observed in Central Asia, China, South America, and Africa (2). Dogs get infected mainly through ingestion of organs (such as the livers and lungs of animals) with fertile cysts. Because of a polluted environment or intimate contact with infected dogs, humans often acquire the infection by accidentally swallowing the parasite's eggs. This parasite can survive in the hosts (including human beings and many animals) for decades without obvious clinical symptoms, which is partially due to the perfect strategies of immune regulation (3). Exploration of these immunological mechanisms may facilitate the development of several novel therapies for the disease.

B cells are in charge of generating protective antibodies after differentiating into antibody-secreting cells in the humoral immune response (4). During the last decade, a population of suppressor B cells, collectively named regulatory B cells (Bregs), has been demonstrated to be associated with the suppression of excessive inflammation (5). Bregs are capable of helping to maintain immunological tolerance. It can limit the immunopathology by producing cytokines such as IL-10, IL-35, and TGF-β, which prevent the proliferation of pathogenic T cells and other pro-inflammatory lymphocytes (6). Several studies have reported that Bregs can be induced by the infection of parasites such as *Leishmania major* and *Schistosoma japonicum* (7–9). We also showed the accumulation of Bregs after the infection of larval *E. granulosus* in mice (10). Given the strong immunosuppressive function, Bregs are thought to be a major immunomodulator in anti-infective immunity. However, how parasitic infection reprograms the function of B cells has yet been identified.

Immunometabolism is a burgeoning field that aims to explore the contribution of key metabolic pathways to immune cell development, differentiation, and function. Accumulating studies have uncovered those metabolic pathways, such as glycolysis, fatty acid oxidation, fatty acid synthesis, and glutaminolysis, that can preferentially determine immune cells' destiny and action (11–14). This phenomenon is due to the lack of large nutrient stores in immune cells, and these effector reactions can only be sustainable when immune cells can dramatically improve their uptake of glucose, fatty acids, and amino acids from their microenvironment (15). On the one hand, the increased uptake of nutrients can provide the substrates for adenosine triphosphate (ATP) synthesis, allowing activated immune cells to maintain their numerous cellular programs. On the other hand, it offers the raw materials for the production of macromolecules like RNA, DNA, proteins, and membranes, which are required for immune cell proliferation and activation. For example, the intrinsic fatty acid reprogramming within immune cells is demonstrated to regulate the outcome of immune response (16, 17). In addition, there is evidence that metabolic reprogramming commits differentiation of human CD27^+^IgD^+^ B cells to plasmablasts or CD27^−^IgD^−^ B cells (18). Thus, in response to extracellular signals, a critical step in the maturation of immune cells is the reprogramming of their cellular metabolism. However, it is still unknown if metabolic reprogramming occurs in B cells infected with the larval stage of *E. granulosus*.

The present study aimed to investigate the specific metabolic reprogramming events associated with the regulatory function of splenic B cells in the mice infected by the larval *E. granulosus*. Using the RNA sequencing technology, a total of 413 differentiated expressed genes (DEGs) (including 303 up- and 110 down-regulated DEGs) were identified after infection. Interestingly, most upregulated DEGs after infection were related to inflammation and chemotaxis, which was accompanied by the elevated expression of key regulators in lipid synthesis and catabolism. Furthermore, a complex network was observed in the DEGs associated with immune and lipid metabolism. Overall, the present study shows that the larval *E. granulosus* infection induces metabolic reprogramming in B cells, which provides a novel clue for clarifying the underlying mechanism of B cell differentiation in parasitic infection.

## Materials and Methods

### Mice, Parasites, Infection

Female C57BL/6J mice (aged 6–8 weeks) were purchased from Shanghai Laboratory Animal Center (SLAC, Shanghai, China) and raised at Xuzhou Medical University's Experimental Animal Center. The mice were randomly assigned into *E. granulosus* group (Eg group) and control group, with 15 mice in each group. The protoscoleces (PSCs) of *E. granulosus* (EgPSC) were acquired by puncture of fertile sheep hydatid cysts in aseptic conditions. The mouse model of larval *E. granulosus* infection was established based on the previous studies (3, 19, 20). The Eg model was established by intraperitoneal injection of 200 μl saline solution containing 2000 live EgPSC for each mouse, and the control mice received 200 μl saline solution. All mice were sacrificed after 6 months after infection. In all our studies, a successful infection is judged by the existence of cysts in the inner organs or abdominal cavity of mice.

### B Cell Isolation

The mouse CD19^+^ B cell isolation kit (Miltenyi, Bergisch Gladbach, Germany) was applied to negatively sort CD19^+^ B cells from the spleens of control and Eg mice. The purity of cells identified via flow cytometry was routinely > 90%. *In vitro* cultivation and RNA sequencing were further performed on these isolated B cells.

### Library Construction and Sequencing

For each sample, approximately 1 × 10^6^ splenic B cells were binned. Each group included three samples from three individual control or infected mice. After total RNA was extracted, eukaryotic mRNA was enriched by Oligo(dT) beads, while prokaryotic mRNA was enriched by removing rRNA by Ribo-Zero^TM^ Magnetic Kit (Epicenter). The enriched mRNA was then fragmented into short fragments using fragmentation buffer and reverse transcripted into cDNA with random primers. Second-strand cDNA was synthesized by DNA polymerase I, RNase H, dNTP, and buffer. Then the cDNA fragments were purified with QiaQuick PCR extraction kit, end-repaired, poly(A) added, and ligated to Illumina sequencing adapters. The ligation products were size selected by agarose gel electrophoresis, PCR amplified, and sequenced using Illumina HiSeq^TM^ 2500 platform by Gene Denovo Biotechnology Co. (Guangzhou, China). The length of pair-end reads was 150 bp.

### Bioinformatics Analysis

The original image data obtained by sequencing was converted into sequence data by Base Calling, which was called raw data or raw reads. The results were stored in FASTQ format, including the sequences of reads and the sequencing quality of bases. To ensure the quality of data, quality control and filtering of data were processed through software fastp (version 0.12.4). Clean data (clean reads) were obtained by removing reads containing adapter, reads containing poly-N, and low-quality reads from raw data. Short reads alignment tool Bowtie2 was used for mapping reads to the ribosome RNA (rRNA) database (21). The rRNA mapped reads will be removed. The rRNA removed reads of each sample were then mapped to the reference genome by HISAT2 (version 2.1.0) (22), respectively.

Gene abundances were quantified by software RSEM (23). The gene expression level was normalized by using the Fragments Per Kilobase of transcript per Million mapped reads (FPKM) method. The FPKM method can eliminate the impacts of different gene lengths and sequencing depth amount on the calculation of gene expression. Therefore, the calculated gene expression can be directly used for comparing the difference of gene expression among samples.

To identify differentially expressed genes between the two groups, the edgeR package (http://www.rproject.org/) was used. We identified genes with |log2FC| > 1 and a false discovery rate (FDR) <0.05 in a comparison as significant DEGs. DEGs were then subjected to enrichment analysis of GO functions and KEGG pathways.

The biological function of differentially expressed mRNAs was investigated by gene ontology (GO) analysis with terms involving biological processes (BP), cellular components (CC), and molecular functions (MF). All DEGs were mapped to GO terms in the GO database [GO.db.3.8.2 (2019/04/26)], gene numbers were calculated for every term, significantly enriched GO terms in DEGs compared to the genome background were defined by hypergeometric test. The calculated *P*-value was gone through FDR Correction, taking FDR ≤ 0.05 as a threshold. GO terms meeting this condition were defined as significantly enriched GO terms in DEGs. The Kyoto Encyclopedia of Genes and Genomes (KEGG) is the major public pathway-related database (Release 94), which was used to identified significantly enriched metabolic pathways or signal transduction pathways in DEGs. The calculated *P*-value was gone through FDR Correction, taking FDR ≤ 0.05 as a threshold. Pathways meeting this condition were defined as significantly enriched pathways in DEG.

### Co-expression Network Analysis

The co-expression network of mRNAs and protein-coding genes was analyzed with Cytoscape (version 3.8.0). Correlations with *P* <0.05 were considered to be statistically significant.

### Validation of Transcriptomic Data Using qRT-PCR

Four genes were chosen randomly for qRT-PCR analysis to obtain further validation of RNA-seq results. Total RNA was isolated from CD19^+^ B cells using TRIzol reagent, and cDNA was synthesized from the RNA using PrimeScript™RT Master Mix. Quantitative PCR analyses were performed in a LightCycler^®^ 480II detection system (Roche Applied Science, Penzberg, Germany) under the following thermal cycler conditions: one cycle of 5 min denaturation at 95°C and then 30 s at 95°C, 30 s at 60°C and 30 s at 72°C for 45 cycles. All experiments were carried out three times and the relative expression of related genes was represented by comparing cycling threshold (Ct) values, which were normalized relative to the endogenous reference (β-actin) on the basis of the 2^−Δ*ΔCt*^ method. The primer sequences used in this study were listed in Table 1.

**Table 1:** The qRT-PCR primer sequences used in the study.

**Primer names**	**Sequences (5^**′**^to 3^**′**^)**
mt-Nd6	Forward: 5′-AGTTCATTATTTTTGGTTG-3′
	Reverse: 5′-TCTCTGGATATTCCTCAGT-3′
Wfdc17	Forward: 5′-CAAATCCATACCTCCCAAC-3′
	Reverse: 5′-TGTCCTTCCTTCTTCTTCC-3′
S100a9	Forward: 5′-CAGCATAACCACCATCATC-3′
	Reverse: 5′-CTCTTCTCTCACAAGCCAA-3′
Gimap4	Forward: 5′-TCAGAGAAGGTCAAAGG-3′
	Reverse: 5′-ATTATCAGGCTGGAAAC-3′
β-Actin	Forward: 5′-CGTGGGCCGCCCTAGGCACCA-3′
	Reverse: 5′-TTGGCCTTAGGGTTCAGGGGGG-3′

## Results

### Identification and Validation of DEGs in Splenic B Cells Post Larval *E. granulosus* Infection

**Figure 1:**
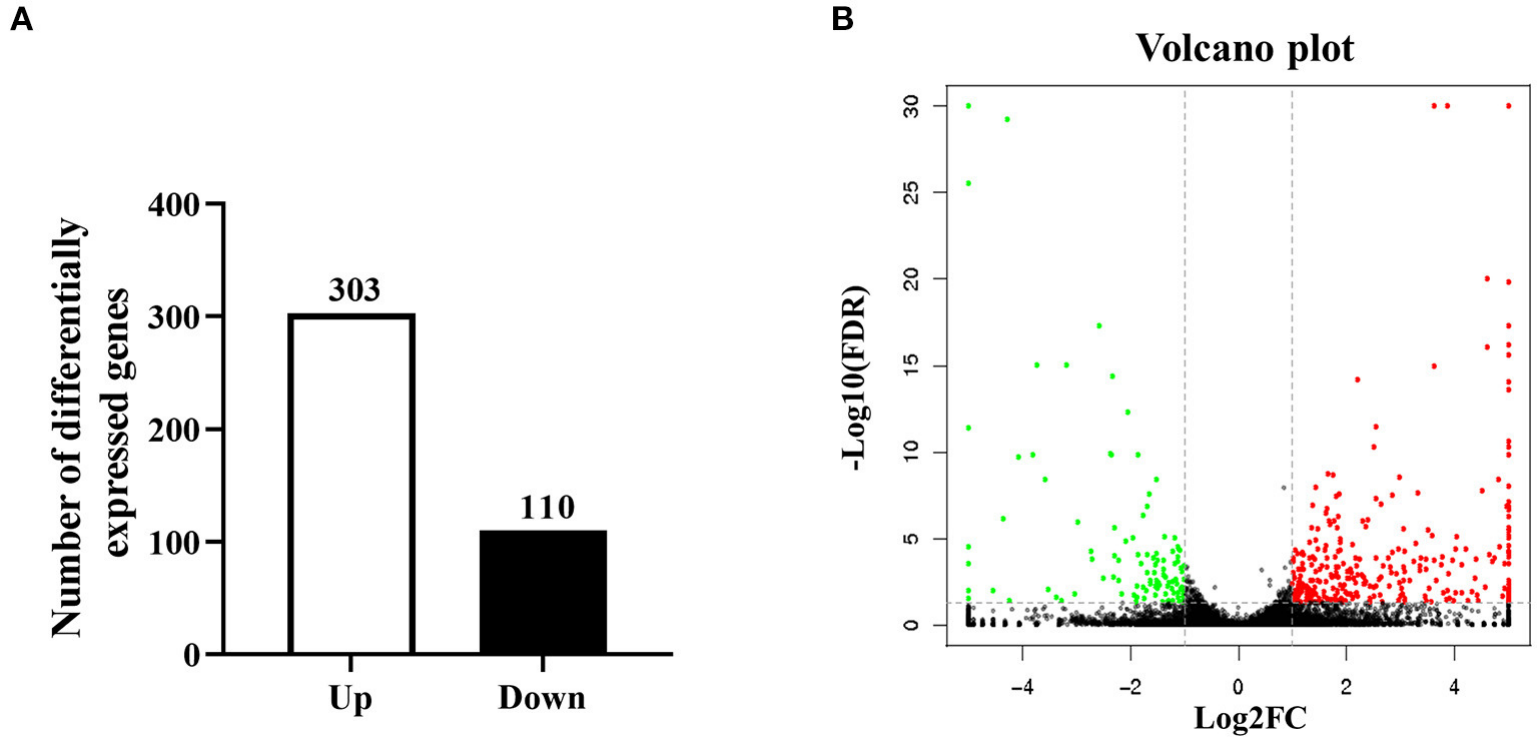
Bioinformatics analysis of DEGs in splenic B cells infected with the larval *E. granulosus*. **(A)** The number of the DEGs. **(B)** The volcano plot shows the distribution of DEGs. *n* = 3 mice per group. The significantly up- and down-regulated RNAs are presented as red or green dots (|log2FC| > 1 and FDR < 0.05), respectively, while the expression of mRNAs not significantly differently expressed is presented as black dots.

B cells represent important regulatory cells that mediate the anti-parasite immune response (24). Our previous studies have shown that larval *E. granulosus* infection induces function changes of B cell function, and notably, numerous differential metabolites were identified in splenic B cells of infected mice (25). To further investigate the specific downstream mechanism of *E. granulosus* on B cell function, total CD19^+^ B cells in spleens were collected from Eg infected and control mice for the RNA sequencing analysis. In order to ensure data quality, it is necessary to quality control and filter the data before information analysis. We further filtered the clean data (clean reads) obtained after the initial filtering to obtain high-quality (HQ) clean data (clean reads) for subsequent information analysis. Q30 percentages of HQ clean data for all samples were higher than 92.88%, and the GC contents of the HQ clean data for all samples ranged between 46.70 and 48.34% (Supplementary Table S1). For further analysis, the HQ clean reads were mapped to the reference genome. Approximately 82.86 to 84.76% of the reads were successfully mapped to the reference genome, and 82.26–84.13% of the reads were uniquely mapped to the reference genome (Supplementary Table S2). All obtained transcriptome data were stored in the SRA database under the number PRJNA726828. The |log2FC| > 1 and FDR <0.05 were considered as the standard to identify DEGs. As shown in Figures 1A,B, a total of 413 DEGs were screened out, including 303 up-regulated and 110 down-regulated DEGs. Hierarchical clustering analysis revealed significant differences in mRNA expression patterns between Eg and control groups.

**Figure 2:**
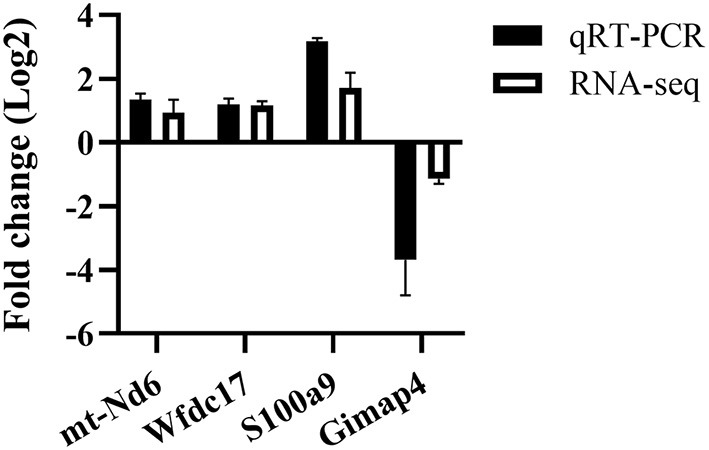
qRT-PCR validation of randomly selected DEGs from the RNA-seq data. *n* = 3–5 mice per group.

For validating the RNA-Seq data, four DEGs (mt-Nd6, Wfdc17, S100a9, Gimap4) were randomly selected for qRT-PCR. Overall, the target gene regulatory direction and expression level differences measured by RT-qPCR were in agreement with the RNA sequencing results (Figure 2), which suggested the data obtained were accurate and reliable.

### GO Analysis of DEGs in Splenic B Cells Post Larval *E. granulosus* Infection

**Figure 3:**
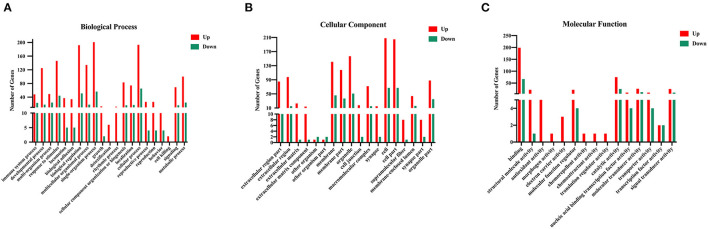
Gene Ontology (GO) analysis of the DEGs in the splenic B cells infected with the larval *E. granulosus*. GO annotation of DEGs with remarkable enrichment scores covering domains of **(A)** biological processes, **(B)** cellular components, and **(C)** molecular functions. The GO terms with FDR (corrected *P*-value) ≤ 0.05 were considered significant.

To investigate the underlying biological functions of DEGs between Eg and control groups, the GO enrichment analysis was executed. GO analysis is commonly used to annotate the physiological functions of a huge number of discovered genes. The enriched GO terms were ordered based on the three categories, including biological processes (BP), cellular components (CC), and molecular function (MF). Three hundred and three upregulated and 110 downregulated DEGs were assigned to 54 and 45 GO terms, respectively. These DEGs were mainly involved in “biological regulation (GO:0065007),” “metabolic process (GO:0008152),” “signaling (GO:0023052),” “immune system process (GO:0002376),” “cell (GO:0043657),” “catalytic activity (GO:0003824)” and “signal transducer activity (GO:0004871)” (Figure 3). These enrichment results could lay the foundation for further exploring the specific mechanisms by which *E. granulosus* regulates the function and differentiation of B cells.

### KEGG Pathway of DEGs in Splenic B Cells Post Larval *E. granulosus* Infection

**Figure 4:**
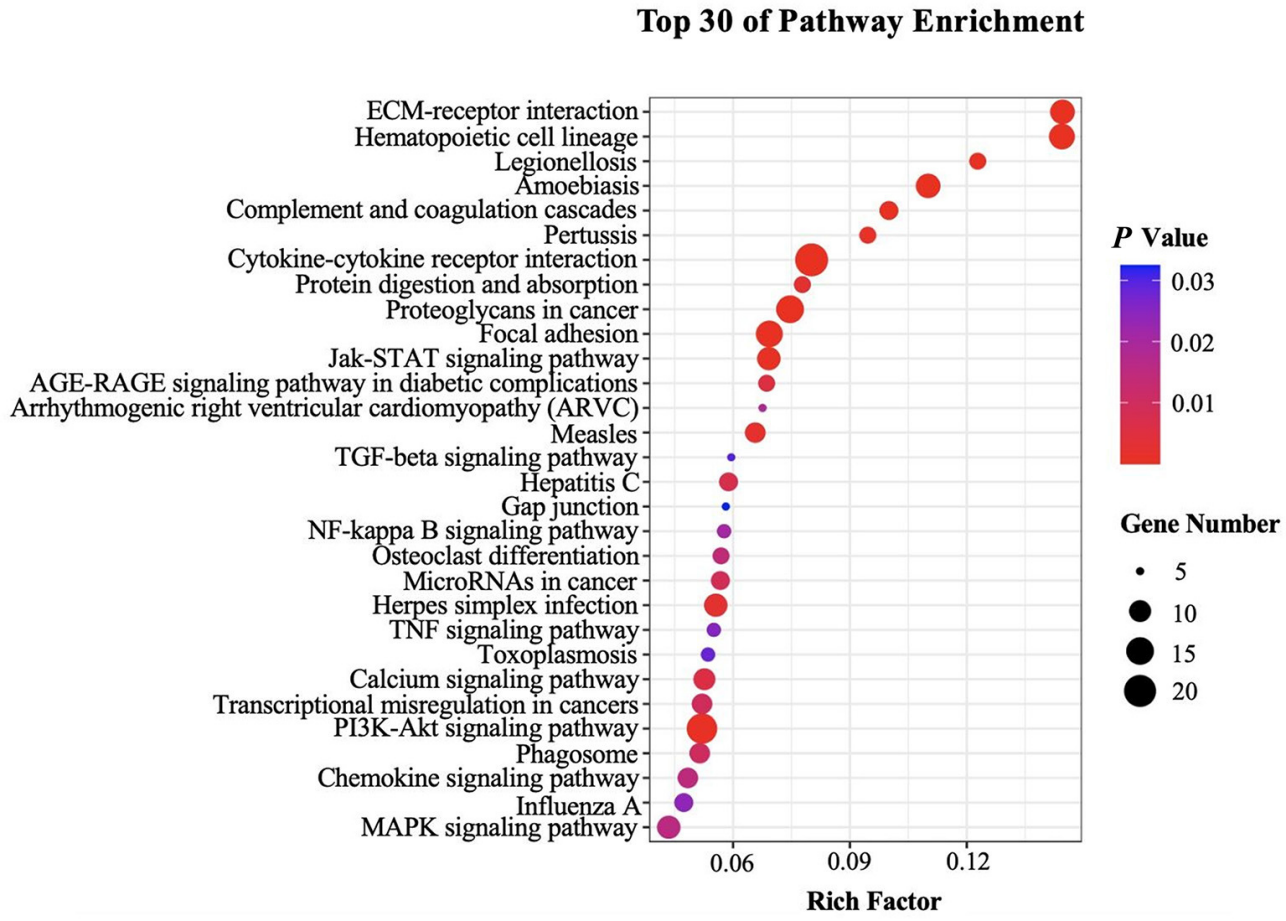
Kyoto Encyclopedia of Genes and Genomes (KEGG) analysis (top 30) of the DEGs in the splenic B cells infected with the larval *E. granulosus*. The size of each point represents the number of DEGs. The larger the point, the more genes fall into this pathway. Moreover, the point of different colors represents the different *P*-values, and the greener point means higher significance of enrichment. The rich factor indicates the degree of enrichment.

Genes in an organism perform their biological functions in a coordinated manner. To further evaluate the significant DEGs related to B cell function and better understand the biological functions of these DEGs, we further carried out KEGG pathway enrichment analysis. DEGs in splenic B cells after infection were mapped to KEGG reference pathways and allocated to 197 pathways. The top 30 enriched pathways were shown in Figure 4. Among them, “Cytokine-cytokine receptor interaction (ko04060),” “ECM-receptor interaction (ko04512),” “PI3K-AKT signaling pathway (ko04151)” and “JAK-STAT signaling pathway (ko04630)” were significantly enriched (Table 2). These pathways were thought to be closely associated with the differentiation and function of B cells after larval *E. granulosus* infection and deserved further study.

**Table 2:** The KEGG pathways of differentially expressed mRNAs.

**Pathway**	**DEGs genes with pathway annotation**	***P*-value**	**Q-value**	**Pathway ID**	**Genes**
**Immune system**					
Hematopoietic cell lineage	13	3.24E-08	6.38E-06	ko04640	Ighg1;Il5ra;Cd34;Il1r1;Il1r2;Cd55;Gp9;Cd9; Itga2b;Cd1d2;Gp1bb;Cd14
Complement and coagulation cascades	8	0.000231228	5.69E-03	ko04610	Cd46;Serping1;C3;Cfh;Cd55;F5;Plat;C1s1
**Signal transduction**					
PI3K-AKT signaling pathway	18	0.000269776	5.91E-03	ko04151	Ighg1;Col6a1;Col1a1;Lama4;Egfr;Col6a2; Osmr;Fn1;Itgav;Pdgfra;Col1a2;Fgfr2;Gng11; Itga2b;Thbs1;Ghr;Col6a5
JAK-STAT signaling pathway	11	0.000436539	8.60E-03	ko04630	Il5ra;Il27ra;Mpl;Osmr;Fhl1;Stat1;Il12a;Il20rb;Lifr; Ghr;Lepr
Calcium signaling pathway	10	0.005951313	7.33E-02	ko04020	Ighg1;Plcg1;Egfr;Pde1b;Htr7;Cacna1s;Pdgfra; Adrb1;Ptger3
**Signaling molecules and interaction**					
Cytokine-cytokine receptor interaction	21	8.46E-08	7.28E-06	ko04060	Il5ra;Mpl;Egfr;Osmr;Cxcl13;Il1r1;Il1r2;Il12a; Pdgfra;Cxcl5;Pf4;Cxcl1;Ccl7;Ccl2;Il20rb; Acvr2a;Lifr;Ghr;Lepr;Cxcl2;Tnfrsf11b
ECM-receptor interaction	12	1.11E-07	7.28E-06	ko04512	Col6a1;Col1a1;Lama4;Col6a2;Fn1;Itgav; Col1a2;Gp9;Itga2b;Thbs1;Gp1bb;Col6a5

### Inflammatory Profile of Splenic B Cells Post Larval *E. granulosus* Infection

**Figure 5:**
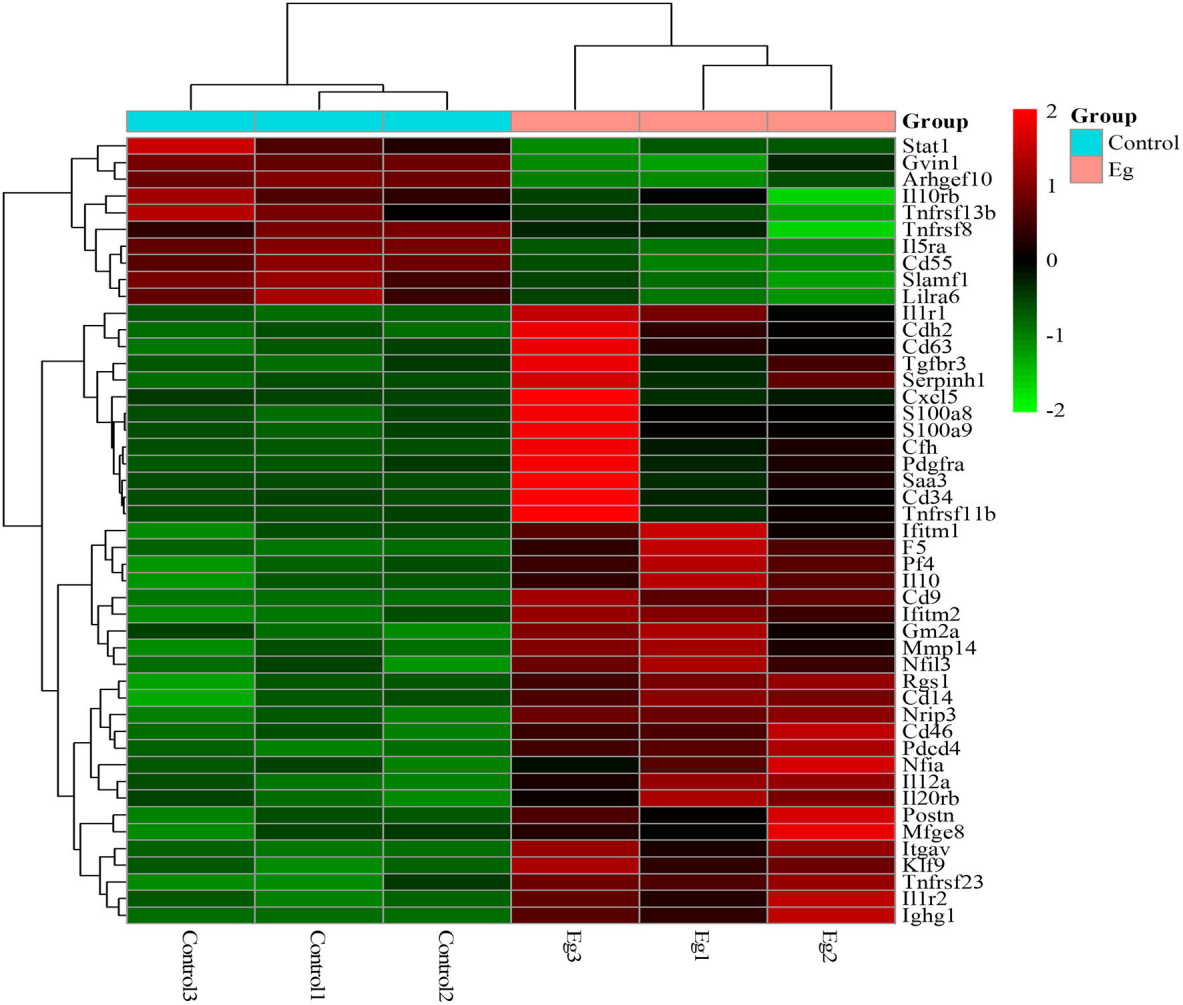
The heatmap of DEGs related to inflammatory signaling molecules, cytokine, and chemokines in the splenic B cells infected with the larval *E. granulosus*. Red color means the upregulated mRNAs and green color means the down-regulated mRNAs.

Cytokines are high-inducible secreted proteins that act as bridges for intercellular communication within the immune system (26). To characterize the cytokine profile of splenic B cells after infection, we analyzed the clustering heatmap of immune-related DEGs between the two groups. As shown in Figure 5, with the infection of larval *E. granulosus*, there were higher expression levels of many inflammatory factors in splenic B cells, such as Cxcl5, Il1r1, S100a8, S100a9, and CD14, which form a complex network of immune regulation. Notably, IL-10 was found to be expressed at a high level. Several cytokines were expressed at low levels, including stat1, Gvin1, Arhgef10, Il10rb, Tnfrsf11b, Tnfrsf8 Il5ra, CD55, Slamf1and Lilra6. It has been reported that down-regulation of Il5ra inhibits TNF-α induced inflammatory response in human nucleus pulposus cells (27). Furthermore, most DEGs related to inflammation and chemotaxis were significantly upregulated after infection. These results were consistent with our previous results that LPS stimulates infected B cells to produce both high levels of pro-inflammatory and anti-inflammatory cytokines (25).

### Metabolic Events of Splenic B Cells Post Larval *E. granulosus* Infection

**Figure 6:**
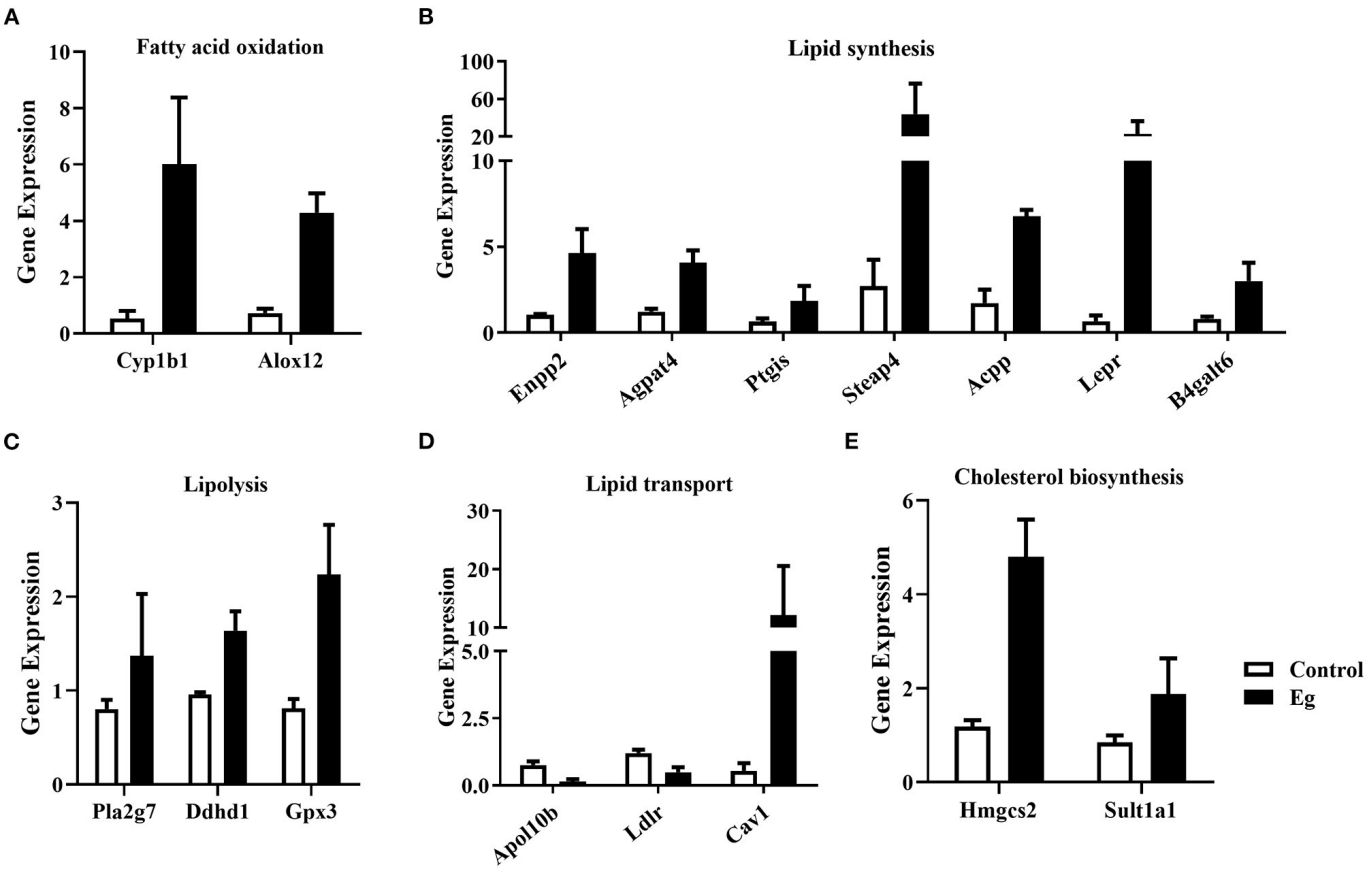
The expression profile of the key genes involved in lipid metabolism in the splenic B cells infected with the larval *E. granulosus*. The expression levels of genes related to fatty acid oxidation **(A)**, lipid synthesis **(B)**, lipolysis **(C)**, lipid transport **(D)**, and cholesterol biosynthesis **(E)** were shown. *n* = 3 mice per group. Cyp1b1, cytochrome P450, family 1, subfamily b, polypeptide 1; Alox12, arachidonate 12-lipoxygenase; Enpp2, ectonucleotide pyrophosphatase/phosphodiesterase 2; Agpat4, 1-acylglycerol-3-phosphate O-acyltransferase 4; Ptgis, prostaglandin I2 (prostacyclin) synthase; Steap4, STEAP family member 4; Acpp, acid phosphatase, prostate; Lepr, leptin receptor; B4galt6, UDP-Gal:beta GlcNAc beta 1,4-galactosyltransferase, polypeptide 6; Pla2g7, phospholipase A2, group VII (platelet-activating factor acetylhydrolase, plasma); Ddhd1, DDHD domain containing 1; Gpx3, glutathione peroxidase 3; Apol10b, apolipoprotein L 10b; Ldlr, low density lipoprotein receptor; Cav1, caveolin 1, caveolae protein; Hmgcs2, 3-hydroxy-3-methylglutaryl-Coenzyme A synthase 2; Sult1a1, sulfotransferase family 1A, phenol-preferring, member 1.

Growing evidence suggests that specific metabolic adaptations are required to allow B cells to develop and differentiate in various environments (28). In this study, these key genes related to fatty acid oxidation (Cyp1b1, Alox12, Figure 6A), lipid synthesis (Enpp2, Agpat4, Ptgis, Steap4, Acpp, Lepr, B4galt6, Figure 6B), lipolysis (Pla2g7, Ddhd1, Gpx3, Figure 6C), lipid transport (Apol10b, Ldlr, Cav1, Figure 6D), cholesterol biosynthesis (Hmgcs2, Sult1a1, Figure 6E) were significantly upregulated. Lipid metabolism plays a crucial role in the function of immunocytes (29). Agpat4/LPA axis in colorectal cancer cells has been validated to regulate p38/p65 signaling-dependent macrophage polarization (30). Besides, in our previous study, 13 differential metabolites involved in lipid metabolism were identified in splenic B cells upon larval *E. granulosus* infection (25). These results indicated that larval *E. granulosus* infection can reprogram lipid metabolism in B cells, thereby modulating its immune function.

### Co-expression Network of Immune and Metabolism Associated DEGs in Splenic B Cells Post Larval *E. granulosus* Infection

**Figure 7:**
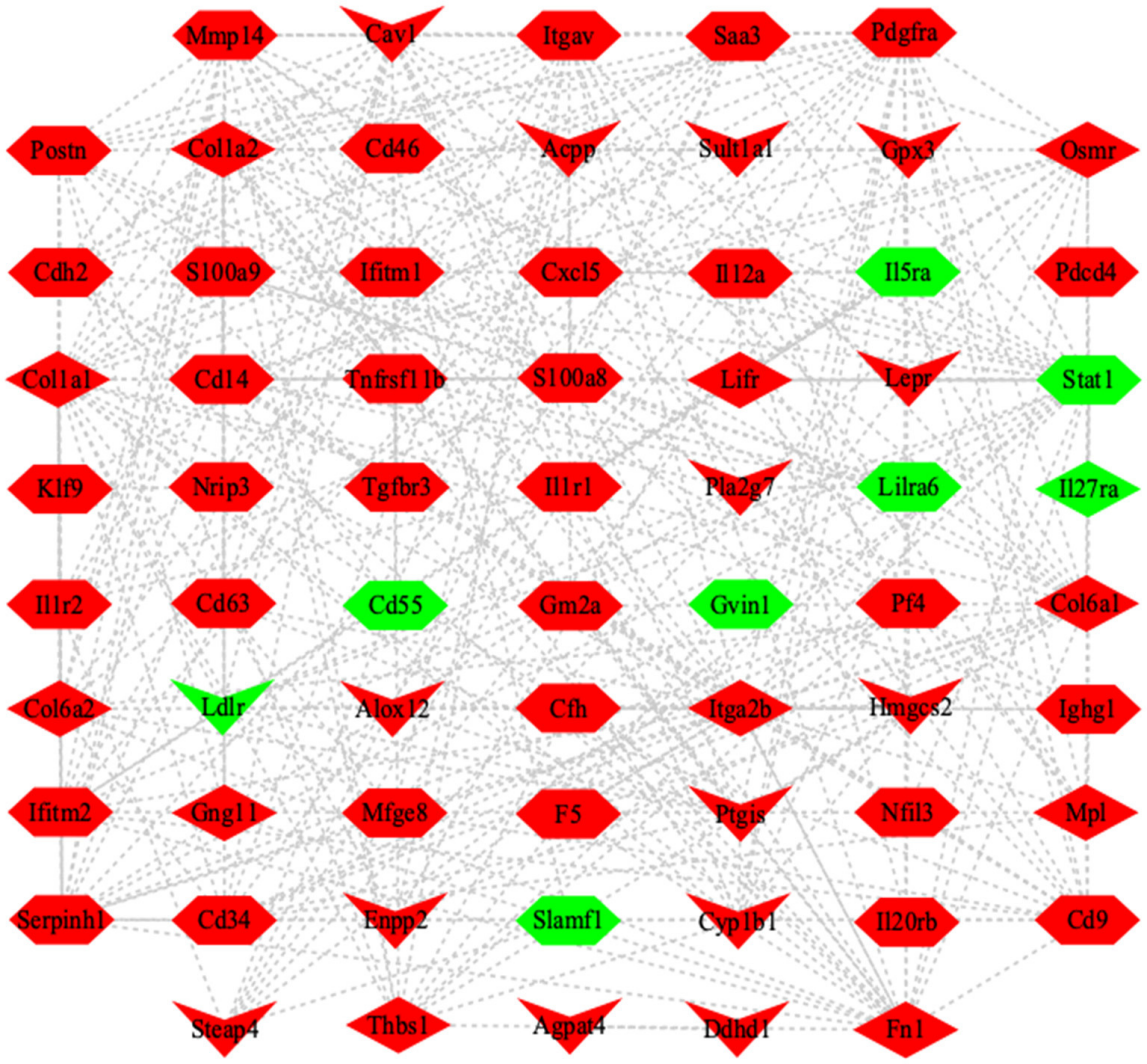
Co-expression network of DEGs in the splenic B cells infected with the larval *E. granulosus*. The network was based on the mathematical relevance (Correlation > 0.99, Correlation < −0.99, and *P*-value < 0.05) to search similar expression profiles of mRNAs using Cytoscape software (version 3.8.0). The hexagon represents inflammation-related genes. V represents the genes related to lipid metabolism. The diamond represents the DEGs in JAK-STAT signaling pathway and PI3K-AKT signaling pathway. Red and green indicate the upregulated and downregulated genes, respectively. The gray line represents the edge that interacts between genes.

Signal transduction and metabolic pathways work together to determine cellular outcomes in an integrated network (28). To reveal the correlation of DEGs among cytokine, lipid metabolism enzyme, and signaling pathway, the co-expression network was built based on mathematical correlation (Correlation > 0.99, Correlation < −0.99, and *P*-value <0.05). The co-expression network was constructed by using Cytoscape (version 3.8.0) (Figure 7), which indicated that larval *E. granulosus* infection induces complex metabolic reprogramming in B cells.

## Discussion

The present study identified a total of 413 DEGs, including 303 up- and 110 down-regulated genes, in the total splenic B cells in mice chronically infected with larval *E. granulosus*. Most DEGs related to inflammation and chemotaxis were significantly upregulated after infection, especially a higher mRNA expression of key regulators associated with lipid metabolism. Furthermore, intimate interaction between these genes of immune and metabolism was shown by co-expression network analysis. Correspondingly, our recent study also identified lots of differential metabolites associated with the functional differentiation and lipid metabolism in the splenic B cells post the parasitic infection (25). Overall, these findings primarily established the potential functional link of metabolic events and B cells' differentiation and function in response to the long-term infection of the larval *E. granulosus*.

Cysticercosis (CE) is a disease resulting from larvae of *E. granulosus* and is one of the most frequent zoonotic diseases in both developed and developing countries (31). The parasite has developed sophisticated strategies to evade host immune responses (3, 20). There is growing evidence that B cells have a crucial role in the modulation of anti-infectious immune response post parasite infection. B cells producing IL-10 were reported to inhibit type I hypersensitivity in mice with *Leishmania major* infection (7). During *Trypanosoma cruzi* infection, IL-17^+^ producing B cells can drive the inflammatory response and favor host resistance (32). Our previous study found an accumulation of IL-10^+^CD19^+^ B cells post larval *E. granulosus* infection (3). Both the inflammatory cytokines (TNF-α, IL-6) and anti-inflammatory cytokines (IL-10) production were significantly elevated in B cells of infected mice after exposure to LPS (25). These results implicated that the parasitic infection alters B cell function. The present study utilized transcriptomics to further characterize the profiles of infected B cells, which offers a novel clue for investigating the fundamental mechanisms.

Immunometabolism is an emerging field of research that reveals the effects of key metabolic pathways on the proliferation/differentiation and function of immune cells (33). Metabolic reprogramming is well-recognized as the critical event in these processes. For example, metabolic pathways such as fatty acid oxidation, fatty acid synthesis, glycolysis, and glutaminolysis, can preferentially determine immune cells' destiny and effector functions (11–14). It is reported that inflammatory stimulants such as LPS and cytokines, can promote the fatty acid synthesis for M1 type macrophages (34). Moreover, compared with Th1, Th2, and Th17 cells, the expression of genes participating in FAO (including CPT1α) is upregulated in Treg cells (35, 36). Likewise, lipid metabolic pathways are reported to regulate B cell fate and function. Studies have shown that energy generated from extracellularly acquired glucose metabolism is used partially to support *de novo* lipogenesis of splenic B cells in response to LPS stimulation, and fatty acid oxidation *in vivo* and *in vitro* can determine the development and survival of optimal germinal center B cells (37, 38). Particularly, HMG-CoA reductase is reported to be a critical enzyme in the early steps of the cholesterol metabolic pathway, and inhibition of HMG-CoA reductase diminishes the ability of B cells to generate IL-10 at the mRNA and protein levels (39). However, the progress of lipid metabolism in B cell differentiation and function is rare in the context of parasitic infection. We previously identified 13 different metabolites related to lipid metabolism after the larval *E. granulosus* infection (25). Moreover, we observed that glutathione, taurine, and inosine can remodel the immune profile in B cells (25). We herein reported the significantly upregulated expression of key genes associated with lipid metabolism. Consequently, these identified differential metabolites and genes may be pivotal in managing B cell differentiation and function *via* reprogramming metabolic fluxes.

The high expression of lipid metabolism is closely related to the reprogramming progress of B cells infected with larval *E. granulosus*, but the specific regulatory mechanism has not been clarified. Autotaxin (ATX), a lysophospholipase, encoded by ENPP2, was upregulated in our study. Autotaxin (ATX)-mediated hydrolysis of lysophospholipid precursors in the extracellular environment produces lysophosphatidic acid (LPA) species. There is evidence that both inflammation and mineralization of the aortic valve are mediated by ATX (40), and glucose homeostasis and insulin sensitivity in older adults are also associated with serum levels of ATX (41). Moreover, glutathione peroxidase 3 (GPx3), accounting for the main antioxidant activity in the plasma, was upregulated in the infected B cells. Insulin receptor expression in white adipose tissue is correlated positively with Gpx3 expression (42). GPx3 overexpression in adipocytes ameliorates hyperglucose-induced insulin resistance and diminished expression of inflammatory genes, while GPx3 neutralization in adipocytes enhances expression of pro-inflammatory genes (43). However, the role of these identified DEGs in the function or differentiation of splenic B cells post *E. granulosus* infection requires further investigation.

KEGG pathway enrichment analysis forecasts the complicated pathways for a general understanding of changes on B cells after infection This study showed that the top enriched KEGG pathways were “ECM-receptor interaction,” “hematopoietic cell lineage,” “PI3K-AKT signaling pathway,” and “JAK-STAT signaling pathway.” PI3K-AKT signaling pathway is engaged in regulating multiple cellular functions such as transcription, translation, proliferation, growth, and survival (44). Class IA of PI3K is specifically required for the growth of B cells, and it mediated signals that induce the expression of the transcription factor Paired box 5 (Pax5), which is instrumental in commitment and differentiation of B cells by activating central B cell-specific signaling proteins such as SLP-65 and CD19 (45). Besides, a previous study showed that IL-10 production by B cells was activated by cecal bacterial lysate through TLR-2 and PI3K (p110δ subunit) pathways (46). PI3K-AKT signaling pathway is critical for the development of pre-B cells and the maintenance of mature B cells (47). JAK-STAT3 signaling pathway plays a key role in regulating many cellular functions such as cell differentiation and proliferation and is strongly related to inflammation due to its involvement in IL-6-signaling (48–51). For example, the JAK-STAT3 signaling pathway, activated by the binding of IL-6 to gp130, has been reported to participate in the growth and differentiation of B cells into plasma cells (49). Notably, JAK-STAT3 signaling pathway can participate in lipid metabolism. The pathway has been reported to regulate fatty acid β-oxidation, which enhances breast cancer stem cells and cancer chemoresistance (52). JAK kinase is also an activator of the PI3K-AKT signaling pathway, and phosphorylated JAK activates PI3K, which in turn activates its downstream cascade (53). In addition, the extracellular matrix (ECM) is composed of a complex mixture of structural and functional macromolecules. Specific interactions between cells and the ECM are mainly mediated by integrins (54). Cellular activities such as adhesion, migration, differentiation, proliferation, survival, and apoptosis, are controlled directly or indirectly by these interactions (55). This study showed that DEGs were significantly enriched in these pathways, suggesting that they may play a role in B cell expansion and differentiation.

The present study observed extensive alternation of inflammation-related DEGs in B cells post the infection of larval *E. granulosus*. This indicated that B cells may be an important contributor to the expression of cytokines. The laminated layer (LL) is the outer layer of the hydatid cyst (the form of larval *E. granulosus* in intermediate hosts). It was reported that in LPS-treated splenocyte cultures, LL crude extract can elevate the mRNA expression levels of Treg-related cytokines (TGF-β, IL-10) and decrease the mRNA expression levels of pro-inflammatory cytokines (IFN-γ, IL-1β, TNF-α) (56). In line with this finding, another study showed that in the early post-infection phase (3–4 weeks), the Th1-type cytokine profile dominates, and then, the response shifts to a Th2-type cytokine profile (57). Thus, it is proposed that the host immunity including B cell response, is tightly reprogrammed by the number and size of the cysts. Furthermore, what we have to point out is that only female mice were used in this study, which were widely used in other studies and ours related to *E. granulosus* infection (3, 10, 20, 25, 56, 57). There is no evidence of gender differences in regard to the parasitic infection, but the effect of gender can't be excluded. Therefore, future studies should focus on the problem, which may help a better understanding of B cells' role and mechanism in the anti-infective immunity induced by larval *E. granulosus*.

## Conclusions

In summary, the present study revealed the functional alternation along with dramatic lipid metabolic reprogramming of the splenic B cells in the mice infected by larval *E. granulosus*. The DEGs were identified, and Co-expression network analysis indicated an intimate interaction between the genes of immune and metabolism in the intrinsic B cells. These results provide a base for further clarifying the underlying mechanism of B cell differentiation and function in response to the long-term infection of larval *E. granulosus*.

## Data Availability Statement

The datasets presented in this study can be found in online repositories. The names of the repository/repositories and accession number(s) can be found in the article/Supplementary Material.

## Ethics Statement

The animal study was reviewed and approved by Ethics Committee of Xuzhou Medical University.

## Author Contributions

WP, FS, and XY: conceived and designed the experiments. SX, YG, TL, PJ, ZY, YH, DW, and ZS: performed the experiments. SX, YG, ZG, and LF: analyzed the data. HL: contributed reagents, materials and analysis tools. FS, WP, SX, and YG: wrote the manuscript. All authors have read and approved the manuscript.

## Funding

This work was funded by the National Natural Science Foundation of China (Nos. 81871670 and 82002164), the Natural Science Foundation of Jiangsu Province (No. BK20201459), the Priority Academic Program Development of Jiangsu Higher Education Institutions, and the Training Programs of Innovation and Entrepreneurship for College Students in Jiangsu Province (Nos. 202010313077Y, 202010313036Z, and 202010313008). The funders had no role in study design, data collection, analysis, decision to publish, or preparation of the manuscript.

## Conflict of Interest

The authors declare that the research was conducted in the absence of any commercial or financial relationships that could be construed as a potential conflict of interest.

## Publisher's Note

All claims expressed in this article are solely those of the authors and do not necessarily represent those of their affiliated organizations, or those of the publisher, the editors and the reviewers. Any product that may be evaluated in this article, or claim that may be made by its manufacturer, is not guaranteed or endorsed by the publisher.
